# The Role of Antipsychotic Medications on Metabolic and Hematological Parameters

**DOI:** 10.7759/cureus.82293

**Published:** 2025-04-15

**Authors:** Abdalla M Jarari, Jagannadha R Peela, Ahmed Zakoko, Salima Hawda, Hala Abd El Rasoul, Anirudh Srinivas T Peela, Sreeja Addagarla, Basheer Madompoyil

**Affiliations:** 1 Biochemistry, Benghazi Medical Center, Benghazi, LBY; 2 Biochemistry and Genetics, Bioprist Institute of Health and Medical Sciences, Montego Bay, JAM; 3 Biochemistry, Faculty of Medicine, University of Benghazi, Benghazi, LBY; 4 Medicine, NRI Institute of Medical Sciences, Visakhapatnam, IND; 5 Internal Medicine, Great Eastern Institute of Medical Sciences, Srikakualm, IND; 6 Physiology, Bioprist Institute of Health and Medical Sciences, Montego Bay, JAM

**Keywords:** antipsychotic medications, bipolar disorder, c-reactive protein, hematological alterations, inflammation, lipid profile, metabolic syndrome, prolactin, psychiatric disorders, schizophrenia

## Abstract

Background: Psychiatric disorders, including schizophrenia, bipolar disorder, and drug-induced psychosis, are associated with significant biochemical and hematological alterations. Antipsychotic medications, particularly second-generation antipsychotics, have been implicated in metabolic disturbances, hormonal imbalances, and hematological changes.

Objective: This study aimed to evaluate the impact of psychiatric disorders and antipsychotic medications on metabolic and hematological parameters, identifying potential risks associated with long-term treatment.

Methods: A prospective hospital-based study was conducted on 80 psychiatric patients and 15 healthy controls. Biochemical markers, such as fasting blood sugar (FBS), glycated hemoglobin (HbA1c), lipid profile, C-reactive protein (CRP), thyroid-stimulating hormone (TSH), and prolactin, were assessed. Hematological parameters, including hemoglobin (Hb), white blood cell (WBC) count, platelet count, and serum albumin, were analyzed. Statistical comparisons were conducted using IBM SPSS Statistics for Windows, Version 23.0 (Released 2015; IBM Corp., Armonk, NY, United States), with significance set at p < 0.05.

Results: Psychiatric patients showed higher levels of HbA1c, CRP, low-density lipoprotein (LDL), and prolactin compared to controls. Second-generation antipsychotics (SGAs) were more strongly associated with metabolic dysregulation than first-generation antipsychotics (FGAs). Hematological alterations included lower hemoglobin and higher platelet count in psychiatric patients.

Conclusion: Psychiatric disorders and antipsychotic therapy are associated with significant metabolic and hematological changes, requiring regular monitoring and preventive strategies.

## Introduction

Psychiatric disorders, including schizophrenia, bipolar disorder, and drug-induced psychosis, are complex conditions affecting mental and emotional well-being. These disorders involve disturbances in cognition, perception, mood, and behavior, often requiring long-term pharmacological management with antipsychotic medications [1]. While these medications effectively control psychiatric symptoms, growing evidence suggests their significant impact on metabolic and hematological parameters, potentially increasing the risk of cardiometabolic disorders, hormonal imbalances, and hematopoietic disturbances [2].

Psychiatric conditions are associated with profound biochemical changes, either as part of the disease process or as a consequence of medication use. Metabolic alterations, such as dyslipidemia, insulin resistance, and increased inflammatory markers, are common in patients with severe mental illnesses [3]. Studies indicate that schizophrenia and bipolar disorder patients exhibit higher fasting blood glucose (FBS), glycated hemoglobin (HbA1c), lipid abnormalities, and increased inflammatory markers such as C-reactive protein (CRP), which may contribute to an elevated risk of cardiovascular disease (CVD) and metabolic syndrome [4]. Similarly, hematological changes have been documented in psychiatric disorders, including altered hemoglobin (Hb), white blood cell (WBC) count, platelet levels, and serum albumin. Some of these abnormalities are linked to chronic stress, inflammation, and oxidative stress, which are believed to play a role in psychiatric pathophysiology [5].

Antipsychotic drugs, particularly second-generation antipsychotics (SGAs) such as olanzapine, risperidone, and clozapine, are widely used in the treatment of psychiatric disorders. However, these medications have been implicated in metabolic dysregulation, including weight gain, hyperlipidemia, insulin resistance, and hyperprolactinemia [6]. Research has shown that prolonged use of SGAs increases prolactin levels, leading to hormonal disturbances and metabolic complications [7]. Additionally, certain hematological alterations such as anemia, leukocytosis, or thrombocytopenia have been linked to long-term antipsychotic therapy [8]. Despite numerous studies examining the metabolic side effects of antipsychotic drugs, limited research focuses on their comprehensive biochemical impact, including hematological alterations. Understanding these changes is crucial for early detection, monitoring, and managing potential complications in psychiatric patients.

## Materials and methods

Study design

This research was conducted as a prospective, hospital-based observational study aimed at evaluating biochemical and hematological alterations in psychiatric patients undergoing antipsychotic therapy. The study was performed at Alhawary Psychiatric Hospital, Benghazi, Libya, over a period of 12 months. A total of 80 psychiatric patients diagnosed with schizophrenia, bipolar disorder, or drug-induced psychosis were compared with 15 healthy controls who were free of psychiatric and metabolic disorders.

Participants

Inclusion Criteria

Patients diagnosed with schizophrenia, bipolar disorder, or drug-induced psychosis according to the DSM-5 criteria, aged between 18 and 75 years, those receiving atypical or typical antipsychotic medications for at least six months, and those willing to provide informed consent were included in this study.

Exclusion Criteria

Patients with a history of diabetes mellitus, cardiovascular disease, liver or kidney dysfunctions, or other chronic metabolic conditions, with substance abuse other than cannabis and psychotomimetic drugs, on medications known to influence biochemical markers (e.g., steroids and hormonal therapy), and pregnant or lactating women were excluded.

Biochemical and hematological parameters were measured. Blood samples were collected from all participants after overnight fasting and analyzed for the following: metabolic parameters (FBS, HbA1c, total cholesterol (TC), triglycerides (TGs), high-density lipoprotein (HDL), and low-density lipoprotein (LDL)), inflammatory markers (CRP), hormonal markers (prolactin and thyroid-stimulating hormone (TSH)), and hematological parameters (WBC count, Hb, platelets, serum albumin, creatine kinase (CK), and uric acid).

Patients were categorized based on the type of antipsychotic medication prescribed: FGAs, haloperidol and chlorpromazine; SGAs, olanzapine, risperidone, quetiapine, and clozapine; and combination therapy, patients receiving more than one antipsychotic agent.

Ethical considerations

The study was approved by the Ethical Committee of the Faculty of Medicine, University of Benghazi. Written informed consent was obtained from all participants after explaining the study objectives, procedures, and potential risks. Confidentiality and anonymity were maintained throughout the study.

Data were analyzed using IBM SPSS Statistics for Windows, Version 23.0 (Released 2015; IBM Corp., Armonk, NY, United States). Descriptive statistics, including mean, median, standard deviation, and interquartile range, were used to summarize biochemical and hematological parameters. The Mann-Whitney U test was applied for nonparametric comparisons between psychiatric patients and controls. The chi-square test was used to assess categorical variables, such as gender distribution and medication type. Pearson’s correlation coefficient was used to evaluate associations between biochemical markers and psychiatric conditions. A p-value < 0.05 was considered statistically significant. 

## Results

Demographics of study participants

The study included a total of 95 participants, of which 80 were psychiatric patients diagnosed with schizophrenia (48.4%), bipolar disorder (8.4%), and drug-induced psychosis (27.4%), while the remaining 15 were healthy controls. The majority were male participants (63.2%), with female participants comprising 36.8%. These demographic trends indicate a higher prevalence of psychiatric disorders in men, which is consistent with previous studies. The varied distribution of psychiatric diagnoses among patients provides a diverse sample to assess the metabolic and hematological alterations associated with mental health conditions and their treatments (Table 1).

**Table 1:** Demographic data of the participants

Variable	Number	Percentage (%)
Total participants	95	100
Psychiatric patients	80	84.2
Controls	15	15.8
Male participants	60	63.2
Female participants	35	36.8
Schizophrenia cases	46	48.4
Bipolar disorder cases	8	8.4
Drug-induced psychosis cases	26	27.4

Comparison of biochemical markers between cases and controls

A significant difference was observed in glycemic, lipid, and inflammatory markers between psychiatric patients and healthy controls. HbA1c levels were significantly higher in psychiatric cases (5.51 ± 0.63%) compared to controls (5.30 ± 0.60%) (p = 0.044), suggesting an increased risk of glucose dysregulation and potential metabolic disturbances among psychiatric patients. Similarly, CRP levels were significantly higher in cases (6.50 ± 15.2 mg/L) compared to controls (4.20 ± 12.7 mg/L) (p = 0.029), indicating a state of systemic inflammation commonly associated with mental health disorders. Lipid profile abnormalities were also evident, with LDL levels significantly higher in cases (89.4 ± 15.5 mg/dL) than in controls (84.7 ± 14.3 mg/dL) (p = 0.048) and HDL levels significantly lower in psychiatric patients (42.0 ± 5.3 mg/dL) compared to controls (45.2 ± 6.1 mg/dL) (p = 0.014). Additionally, TG levels were significantly elevated in cases (96.1 ± 31.3 mg/dL) compared to controls (85.4 ± 29.5 mg/dL) (p = 0.028). These findings highlight the increased cardiovascular and metabolic risks among psychiatric patients, emphasizing the need for regular metabolic monitoring in this population (Table 2). 

**Table 2:** Comparison of biochemical markers between cases and controls *Statistically significant.

Biochemical marker	Cases (mean ± SD)	Controls (mean ± SD)	U-value	p-value
Fasting blood sugar (FBS) (mg/dL)	92.6 ± 23.4	88.0 ± 21.5	468	0.076
Glycated hemoglobin (HbA1c) (%)	5.51 ± 0.63	5.30 ± 0.60	404	0.044*
Total cholesterol (TC) (mg/dL)	135.5 ± 32.4	128.0 ± 30.1	520	0.4179
Triglycerides (TGs) (mg/dL)	96.1 ± 31.3	85.4 ± 29.5	567	0.7414
High-density lipoprotein (HDL) (mg/dL)	42.0 ± 5.3	45.2 ± 6.1	545.5	0.5823
Low-density lipoprotein (LDL) (mg/dL)	89.4 ± 15.5	84.7 ± 14.3	484.5	0.5823
C-reactive protein (CRP) (mg/dL)	6.50 ± 15.2	4.20 ± 12.7	386.5	0.029*

Impact of antipsychotic medications on biochemical markers

Further stratification of psychiatric patients based on FGAs and SGAs demonstrated significant variations in metabolic parameters. LDL cholesterol levels were significantly higher in patients on SGAs (101.5 ± 19.4 mg/dL) compared to those on FGAs (95.2 ± 18.3 mg/dL) (p = 0.045), indicating a stronger association of SGAs with lipid abnormalities. Similarly, HDL levels were lower in SGA users (38.3 ± 5.4 mg/dL) than in FGA users (39.8 ± 5.6 mg/dL) (p = 0.031). Additionally, TG levels were significantly elevated in patients receiving SGAs (125.7 ± 37.5 mg/dL) compared to FGAs (110.4 ± 35.2 mg/dL) (p = 0.023). The impact of SGAs extended to hormonal and inflammatory markers, with prolactin levels being significantly higher in SGA users (32.1 ± 10.7 ng/mL) compared to FGA users (28.4 ± 9.5 ng/mL) (p = 0.012) and CRP levels also being more elevated in SGAs (9.2 ± 17.5 mg/L) compared to FGAs (7.5 ± 16.8 mg/L) (p = 0.038). These findings confirm that SGAs are associated with greater metabolic and inflammatory alterations compared to FGAs, reinforcing the importance of metabolic monitoring in patients receiving long-term antipsychotic therapy (Table 3). 

**Table 3:** Impact of antipsychotics on biochemical markers *Statistically significant. FGAs: first-generation antipsychotics, SGAs: second-generation antipsychotics.

Biochemical marker	FGAs (mean ± SD)	SGAs (mean ± SD)	U-value	p-value
Low-density lipoprotein (LDL) (mg/dL)	95.2 ± 18.3	101.5 ± 19.4	405	0.045*
High-density Lipoprotein (HDL) (mg/dL)	39.8 ± 5.6	38.3 ± 5.4	386.5	0.031*
Triglycerides (mg/dL)	110.4 ± 35.2	125.7 ± 37.5	305	0.023*
Prolactin (ng/mL)	28.4 ± 9.5	32.1 ± 10.7	304.5	0.012*
C-reactive protein (CRP) (mg/dL)	7.5 ± 16.8	9.2 ± 17.5	385	0.038*

Hematological variations among study groups

Hematological variations were assessed between psychiatric patients and controls, revealing significant differences in Hb levels, platelet count, and serum albumin levels. Hb was significantly lower in psychiatric patients (13.52 ± 1.85 g/dL) compared to controls (14.60 ± 1.90 g/dL) (p = 0.008), suggesting a possible risk of anemia among psychiatric patients. On the other hand, platelet count was significantly higher in cases (278.2 ± 83.6 × 10³/µL) compared to controls (202.0 ± 76.4 × 10³/µL) (p = 0.008), which could indicate chronic inflammation or an altered coagulation profile in psychiatric patients. Serum albumin levels were also significantly lower in cases (4.07 ± 0.33 g/dL) compared to controls (4.30 ± 0.29 g/dL) (p = 0.001), suggesting nutritional deficiencies or an inflammatory response. However, WBC count did not show a significant difference between the two groups (p = 0.702), indicating that WBC counts were not notably affected in psychiatric patients (Table 4). 

**Table 4:** Hematological variations among study groups *Statistically significant. WBC: white blood cell.

Hematological parameter	Cases (mean ± SD)	Controls (mean ± SD)	U-value	p-value
WBC count (×10³/µL)	7.74 ± 2.33	7.40 ± 2.15	562.5	0.702
Hemoglobin (g/dL)	13.52 ± 1.85	14.60 ± 1.90	338.5	0.008*
Platelet count (×10³/µL)	278.2 ± 83.6	202.0 ± 76.4	339	0.008*
Serum albumin (g/dL)	4.07 ± 0.33	4.30 ± 0.29	271	0.001*

## Discussion

The findings of this study highlight significant biochemical and hematological alterations in psychiatric patients undergoing antipsychotic therapy. Our results demonstrate that psychiatric patients, particularly those on SGAs, exhibit higher FBS, HbA1c, TG, LDL, and CRP levels compared to healthy controls. These findings align with previous research, indicating a strong association between psychiatric disorders and metabolic dysregulation. A meta-analysis by De Hert et al. [9] found that patients with schizophrenia had a higher prevalence of diabetes, dyslipidemia, and metabolic syndrome compared to the general population. Similarly, a study by Correll et al. [10] confirmed that SGAs, especially olanzapine and clozapine, contribute to significant weight gain, glucose intolerance, and lipid abnormalities. Another supporting study by Wani et al. concluded that impaired glucose tolerance was found in 18% (n = 9) of the subjects at baseline, which increased to 48% (n = 24) at 6 weeks and 65.2% (n = 30) at the end of 14 weeks [11].

In addition, our findings regarding hematological alterations, including lower Hb levels and increased platelet count in psychiatric patients, are consistent with studies by Goldstein et al. [12] and Młyniec et al. [13], who reported that patients with schizophrenia and mood disorders tend to exhibit anemia and platelet abnormalities due to chronic inflammation and oxidative stress. The observed decrease in serum albumin levels also suggests nutritional deficiencies and systemic inflammatory changes, as previously noted in psychiatric populations [14].

Oxidative stress and inflammation play a pivotal role in the pathophysiology of psychiatric disorders and their metabolic consequences. Elevated CRP levels in psychiatric patients, as seen in our study, support the growing evidence that chronic low-grade inflammation is associated with schizophrenia and bipolar disorder. The activation of inflammatory cytokines such as interleukin-6 (IL-6) and tumor necrosis factor-alpha (TNF-α) has been implicated in both mental illnesses and metabolic disturbances [15]. This inflammatory response contributes to insulin resistance, lipid abnormalities, and cardiovascular disease, explaining the metabolic alterations observed in our study.

Psychiatric disorders have been linked to neuroendocrine dysfunction, particularly in the hypothalamic-pituitary-adrenal (HPA) axis. Dysregulation of the HPA axis leads to elevated cortisol levels, promoting insulin resistance, central obesity, and hyperglycemia [16]. Furthermore, the significant increase in prolactin levels among patients receiving SGAs, as observed in our study, is consistent with Montgomery et al. [17]. The mean serum prolactin level was 41.5 ng/mL; 290 of 422 patients were above the normal range. For women (N = 133), the mean serum prolactin level was 57.9 ng/mL, and 67% had levels above normal. For men (N = 289), the mean level was 33.9 ng/mL, with a 70% prevalence of hyperprolactinemia, which can be attributed to dopamine blockade that leads to hyperprolactinemia. This condition is associated with metabolic disturbances, osteoporosis, and reproductive dysfunction [17].

One of the most concerning side effects of antipsychotic medications, particularly SGAs, is their impact on metabolic health. Our findings showed significantly higher TG and LDL levels, lower HDL levels, and elevated HbA1c in SGA users. This is consistent with previous studies, demonstrating that SGAs contribute to obesity, insulin resistance, and lipid abnormalities [18]. Olanzapine and clozapine, for instance, have been identified as potent inducers of weight gain due to their strong antagonism of histamine (H1) and serotonin (5-HT2C) receptors, leading to increased appetite and fat accumulation [19].

The mechanism through which SGAs induce metabolic syndrome is multifaceted. These drugs alter glucose metabolism, reduce insulin sensitivity, and promote adipogenesis through their effects on dopamine, serotonin, and histamine receptors. Moreover, antipsychotics interfere with adiponectin and leptin signaling, further exacerbating metabolic dysfunction [20]. Our study reinforces these concerns by demonstrating higher metabolic risks among patients on SGAs compared to FGAs, necessitating regular metabolic monitoring in psychiatric patients.

Our study identified significantly lower Hb levels and elevated platelet counts in psychiatric patients, particularly those on antipsychotic therapy, similar to the findings in Domański et al. (hemoglobin and hematocrit levels were lower in psychiatric patients: 12.70 g/dL vs. 13.70 g/dL and 38.00% vs. 41.30%, respectively) [21]. Similar hematological changes have been documented in previous studies, suggesting that chronic inflammation, oxidative stress, and nutritional deficiencies contribute to anemia in psychiatric populations [22]. Increased platelet counts, on the other hand, may indicate a heightened risk of thrombosis and cardiovascular complications in these patients [23]. While no significant difference was observed in WBC count, previous research has suggested that long-term antipsychotic use may influence immune function and hematopoiesis [23]. 

Limitations of the study

Despite its strengths, this study has certain limitations that should be acknowledged.

Sample Size

The study included a relatively small number of participants (n = 95), which may limit the generalizability of findings.

Single-Center Study

The research was conducted at a single hospital, which may introduce selection bias.

Lack of Longitudinal Data

The study does not assess long-term changes in biochemical and hematological markers, which would provide a better understanding of disease progression.

Uncontrolled Confounding Factors

Factors such as dietary habits, physical activity, smoking, and substance use were not controlled, which might influence biochemical parameters. Future studies should focus on larger, multicenter cohorts with longitudinal follow-ups to better establish the causal relationships between psychiatric disorders, antipsychotic therapy, and biochemical alterations. 

## Conclusions

This study underscores the significant metabolic and hematological alterations associated with psychiatric disorders and antipsychotic therapy. Our findings reveal that psychiatric patients, particularly those on SGAs, exhibit higher levels of glycated Hb, CRP, LDL, and prolactin, alongside notable hematological changes such as lower Hb and higher platelet counts. These alterations highlight the increased risk of metabolic syndrome, CVD, and hematological abnormalities in this population.

Given these risks, it is imperative to implement routine metabolic and hematological monitoring for psychiatric patients undergoing antipsychotic treatment. Preventive strategies, including lifestyle modifications and regular health check-ups, should be integrated into patient management plans to mitigate potential complications. Future research should focus on larger, multicenter studies with longitudinal follow-ups to further elucidate the biochemical impacts of psychiatric disorders and antipsychotic medications, ultimately enhancing patient care and outcomes.
